# Decision Support Tools Strengthen Early Peanut Introduction Practices and Streamline Data Automation

**DOI:** 10.7759/cureus.104769

**Published:** 2026-03-06

**Authors:** Lauren E Herlihy, Cristina Collins, Kelly Reilly, Elizabeth Blyth, Laura Gay, Priyanka Rao, Meaghan Sowers, Katherine Jordan

**Affiliations:** 1 Pediatrics, University of North Carolina at Chapel Hill, Chapel Hill, USA; 2 Institute for Healthcare Quality Improvement, University of North Carolina at Chapel Hill, Chapel Hill, USA; 3 Operational Excellence, University of North Carolina at Chapel Hill, Chapel Hill, USA; 4 Family Medicine, University of North Carolina at Chapel Hill, Chapel Hill, USA; 5 Nursing, University of North Carolina at Chapel Hill, Chapel Hill, USA

**Keywords:** decision-support tools, electronic medical record (emr), peanut allergy, plan-do-study-act (pdsa), prevention in primary care

## Abstract

Objective: Peanut allergy prevalence in children persists in the United States. Early peanut introduction (EPI) is now recognized as an evidence-based guideline for preventing peanut allergy. EPI implementation relies on primary care providers (PCPs) guiding caregivers of young infants as they transition to solid foods. We aimed to increase caregiver-reported peanut consumption among infants at six-, nine-, and 12-month well-child checks (WCCs) across three physician-based outpatient clinics.

Methodology: A quality improvement (QI) team adapted a clinical decision support (CDS) toolkit, initially developed as a pilot project at an academic residency clinic, for use at three additional outpatient clinics with distinct demographic profiles. Spreading the initiative involved developing an automated reporting tool that utilized the health system’s electronic medical record (EMR) dashboard to pull data from provider note templates. The team analyzed data during the intervention and sustainability phases at all clinics using Statistical Process Control Charts (SPCCs).

Results: Data from the automated report showed that caregiver-reported peanut consumption at the six-, nine-, and 12-month WCCs increased from 0 (0%) at baseline to 1,579 (62.8%) post-intervention. Documentation of EPI guidance at four-, six-, and nine-month WCCs increased from 0 (0%) at baseline to 1,667 (96%) post-intervention. Distribution of home peanut introduction handouts at four-, six-, and nine-month WCCs increased from 0 (0%) at baseline to 1,015 (77.9%) post-intervention. The mean in-room time was 48.8 minutes from baseline through sustainability.

Conclusions: Modifying features of the pilot CDS toolkit performed well when spread to more diverse PCP clinics in the same health system. Adding an innovative reporting tool to the EMR dashboard can provide sustainable analytics for QI metrics. Continued partnerships with PCPs and the use of CDS toolkits, along with reporting tools, may improve outcomes for introducing allergenic foods beyond peanuts, an ongoing unmet need in primary care.

## Introduction

Peanut allergy remains a significant public health concern, affecting approximately 2% of children in the United States [1-6]. Over the past decade, early peanut introduction (EPI) has emerged as a preventive strategy, with evidence and guidelines reshaping clinical practices [2,4,7-18]. However, after nearly a decade, implementation in primary care remains less widespread than we would like. The 2015 Learning Early About Peanut Allergy (LEAP) study demonstrated that introducing peanuts between four and 11 months reduces peanut allergy risk in infants with severe eczema or egg allergy [17]. In the years following, professional organizations and consensus groups issued guidance on EPI and other allergenic foods, including updated guidance from the American Academy of Pediatrics (AAP) [1,3,5,8-10,12,13,15,16,19].

The role of primary and secondary prevention of disease typically belongs with primary care providers (PCPs) [20,21]. However, translating allergen introduction guidelines into routine clinical practice remains challenging due to provider knowledge gaps, inconsistent messaging, and parental fears [5,9,12,15,16,22,23]. A follow-up to the LEAP study examined former participants, now six to 13 years old, and concluded that EPI induced long-term tolerance despite variable consumption patterns after age five [18]. For this reason, efforts to standardize EPI practices in primary care settings must persist.

In a pilot quality improvement (QI) initiative at an academic, residency-based continuity clinic in North Carolina, researchers addressed barriers to EPI guidance through standardization [7]. Serving a diverse population, this initiative integrated clinical decision support (CDS) tools and Epic electronic medical record (EMR) standardization into pediatric workflows to improve guideline adherence. The CDS toolkit provided Epic EMR prompts with embedded smart data elements (lists and phrases) in the Epic EMR note templates to guide providers during well-child checks (WCCs) during the first year of life, streamlining risk stratification based on eczema severity and history of egg allergy in a decision tree, and facilitating appropriate peanut introduction. These interventions were associated with improvements in all measured outcomes related to EPI guideline adherence, including increased rates of caregiver-reported peanut consumption by nine months of age, while maintaining balancing measures without special cause variations [7]. CDS tools can improve measured outcomes, including documentation, patient education, and patient outcomes, by bridging gaps between clinical practice and evidence-based guidelines [3,7,24,25].

Access to effective CDS tools enables PCPs to deliver anticipatory guidance on allergen introduction during routine WCCs in the first year of life. This spread QI project builds upon the initial success of the pilot QI initiative, increasing caregiver-reported peanut consumption at a single clinic [7]. This spread project was needed to assess the feasibility of widespread adoption of the EPI CDS toolkits in more diverse clinical settings. The project components include the following: typical QI framework measures (outcome, process, and balancing) related to CDS toolkit use and the rollout of an innovative automated EMR reporting dashboard.

## Materials and methods

Context

This QI spread project targeted infants seen for routine care at four-, six-, nine-, and 12-month WCCs, at non-residency, physician-based clinics, with engagement from the QI team beginning at each clinic’s kickoff session (Clinic 1: November 8, 2023; Clinic 2: December 8, 2023; Clinic 3: February 2, 2024). The team provided all three clinics with the CDS toolkit bundle, including EPIC EMR smart phrases, on a rolling basis for testing and feedback beginning December 20, 2023. And though the QI team provided education, coaching, and support during the toolkit's trial period with providers, the automated data-collection tool was not deployed to live production in the EMR until January 24, 2024. The automated report tracked intervention and sustainability data from then through March 2025.

Although success was achieved during the pilot project at a single academic residency clinic in the year prior [7], the toolkit's effectiveness in physician-based clinics had not been tested and remained unknown. Operationally, to test the robustness of our CDS tools across a broader context, we opted to deploy the toolkit in three different physician-based clinics, mentioned above as Clinic 1, Clinic 2, and Clinic 3, where a mix of physicians and advanced practice providers still had the option to use their individualized note templates and after-visit summaries (AVSs) for patients, as these clinicians were more likely to have an established clinic flow unique to their practice style and preference, unlike the residency clinic, where note templates and AVSs are standardized for teaching purposes.

The selected clinics shared the same Epic EMR and served multiple counties in North Carolina. Table 1 shows the population’s demographics and references the physician-based locations at *Clinic 1*, *Clinic 2*, and *Clinic 3*. Clinics 1 and 3 were pediatric, and Clinic 2 was a family medicine clinic. Structurally, including a busy family medicine-based clinic whose practice had the fewest infant WCCs was essential to assess whether the CDS toolkit could sustain efficacy when individual providers may go days or weeks without engaging with the tools. In summary, by including three additional clinics with diverse patient populations and provider teams, rather than a homogeneous resident clinic, we stress-tested the CDS toolkit while adding innovative enhancements.

**Table 1:** Physician-based clinic demographics (n = 1,206 unique patients). Data are presented as *n* (%). *More individualized demographic fields have been condensed for this table. Each patient is counted only once in this table, although the same patient may appear multiple times in the full dataset if seen during their four-, six-, nine-, and 12-month visits during the analysis period (six weeks before each clinic’s kickoff through March 7, 2025). This table excludes twins, patients likely to be allergic, patients already diagnosed with peanut allergy, and patients who had an allergic reaction after trying peanuts.

	Clinic 1 (Pediatrics), *n* (%)	Clinic 2 (Family Medicine), *n* (%)	Clinic 3 (Pediatrics), *n* (%)	Total, *n* (%)
Gender				
Female	335 (27.8%)	68 (5.6%)	180 (14.9%)	583 (48.3%)
Male	356 (29.5%)	82 (6.8%)	185 (1.5%)	623 (51.7%)
Race				
American Indian/Alaskan Native	1 (0.0%)	1 (0.0%)	7 (0.6%)	9 (0.0%)
Asian*	52 (4.3%)	6 (0.5%)	55 (4.6%)	113 (9.4%)
Black	170 (14.1%)	49 (4.1%)	99 (8.2%)	318 (26.4%)
Other Race*	86 (7.1%)	19 (1.6%)	66 (5.5%)	171 (14.2%)
Prefer not to answer	41 (3.4%)	2 (0.0%)	23 (1.9%)	66 (5.5%)
White	342 (28.4%)	72 (6.0%)	115 (9.5%)	529 (43.9%)
Language				
English	662 (54.9%)	148 (12.3%)	337 (27.9%)	1147 (95.1%)
Spanish	22 (1.8%)	1 (0.0%)	18 (1.5%)	41 (3.4%)
Other*	4 (0.3%)	1 (0.0%)	10 (0.8%)	15 (1.2%)
Unknown	3 (0.2%)	0 (0.0%)	0 (0.0%)	3 (0.2%)
Ethnicity	-	-	-	-
Hispanic or Latino*	93 (7.7%)	23 (1.9%)	66 (5.5%)	182 (15.1%)
Not Hispanic or Latino	559 (46.4%)	126 (10.4%)	279 (23.1%)	964 (80.0%)
Prefer not to answer	39 (3.2%)	1 (0.0%)	20 (1.7%)	60 (5.0%)
Coverage				
Medicaid	212 (17.6%)	67 (5.6%)	160 (13.3%)	439 (36.4%)
Non-Medicaid*	465 (38.6%)	83 (6.9%)	219 (18.2%)	767 (63.6%)

Interventions

The team modified previous CDS tools from the pilot project and added innovative elements to the toolkit. We revised Epic EMR smart lists to improve the planned data sets generated by our new reporting system. New to the project, we added a provider-facing frequently asked questions (FAQ) document about EPI and developed a dashboard tool in the Epic EMR that automated data reporting, allowing the team to collect and analyze data from a larger sample and filter individual variables. The team used plan-do-study-act (PDSA) cycles to achieve project aims.

The project leadership included practice facilitators who coordinated engagement across busy clinics. Each participating clinic identified a provider champion to facilitate communication between clinic stakeholders and the QI team. QI meetings at each site included kickoff and process-mapping sessions. Project leadership provided regular performance feedback to champions during remote web-based conferences. These meetings also enabled champions to relay collective feedback from their participating clinics’ providers, allowing the QI team to make project changes through PDSA cycles. Continued practice facilitation increased adherence to the evidence-based guidelines and the success of the intervention [26,27].

The previously published work protocol was adapted to accommodate variation in practice among all three participating clinics [7]. While the pilot work protocol guided providers to advise caregivers to introduce peanut products at home for most infants seen in our clinics, the protocol also included decision trees for infants at higher risk of peanut allergy, as risk stratification was a key feature of the addendum guidelines [1]. As the project moved into sustainability, consensus guidelines were updated to recommend against testing before introduction, regardless of risk level [8,28]. Our clinics subsequently phased out pre-introduction testing from the protocol, emphasized early introduction for all infants who were developmentally ready for feeding, and relied on urgent, timely referrals when needed. When clinical history was consistent with an allergic reaction to peanuts, providers were prompted to recommend avoiding peanuts and make a referral to an allergy specialist.

At the six-, nine-, and 12-month visits, the Epic EMR note template smart data elements prompted providers to assess or reassess if patients were consuming peanut products since their last visit and complete the EPI assessment, including prompts to provide the home introduction handout if appropriate. Peanut consumption at the four-month WCCs was not included in the primary outcome because this visit type is when the guidance would have first been introduced by providers.

Home Peanut Introduction Handout

The QI team, following prior work, found that peanut butter was the most accessible peanut-containing food. While completing the peanut consumption questions in the WCC templates, providers were prompted to include an English or Spanish handout in the patient’s AVS for infants. Additional modifications were made based on providers’ direct feedback. The QI team conducted several iterative PDSA cycles to improve reliability and reduce workflow friction. To reduce the number of manual clicks required to insert the handout into the patient’s AVS, the team designed options for Epic EMR users, such as vanishing texts, smart buttons, and strategically placed smart lists to trigger handout insertion. The team also created a video for providers outlining how to customize the home peanut introduction handout in their WCC encounters. Lastly, the team modified the handout from the previous iteration to clarify guidance on the frequency, in addition to the already targeted quantity, of peanut consumption, as evidence supports that frequent and ongoing regular consumption is immunologically important to prevent peanut allergy [28]. 

Donated Supply of Peanut Butter

Concerns raised by some providers during a clinic kickoff meeting included caregivers reporting economic constraints or cultural dietary preferences that limited peanut butter availability at home. In response to this feedback, the team secured a donation to supply peanut butter jars to families with limited access. This intervention aimed to ensure the initiative did not unintentionally widen existing health disparities.

FAQs Document

Similarly, a component of the project developed from direct provider feedback specific to local input and context of this project, the team developed an FAQ document to empower clinic providers to answer caregivers' questions about food allergies and peanut introduction. The patient improvement partner, new to the spread project, provided valuable insights, edits, and suggestions from the family's and child's perspectives, adding depth to this FAQ tool. The tool aims to avoid unnecessary delays in home introduction and avoid allergy referrals when not clinically indicated. The document serves as a provider reference tool and was posted and easily visible in workrooms throughout all the clinics (Table 2). Of note, the handout addressed differences in cultural norms for dietary consumption of peanut butter, with knowledge that non-White infants carry a higher risk of developing peanut allergy as compared to white infants in the first year of life [29].

**Table 2:** Provider-facing FAQ document. A tool to empower PCPs to answer common questions from caregivers about early introduction and food allergies during infant WCCs. FAQ, frequently asked question; PCP, primary care provider; WCC, well-child check

Early peanut introduction in babies		
Questions	Answers	Takeaways/Suggestion
Can I use a pre-packaged allergen kit instead of feeding peanut butter to my baby?	There are many popular products on the market that advertise early allergen introduction. They typically have starter products containing peanuts, milk, and eggs. The packets geared toward older infants often include nine major allergens.	Caregivers can use these products; however, good anticipatory guidance is needed about the quantities and frequency of ingestion. Always remind caregivers *not to use the packets *if the baby has a *known* allergy to one of the allergens in the packets. Using the packets for desensitization therapy for known allergies could be dangerous and lead to allergic reactions.
Someone else in our household is allergic to peanuts. Is it okay to introduce peanuts to my baby?	Yes, with proper precautions for the allergic child. If offering the baby peanut-containing foods, place the child in the highchair to eat. Wipe surfaces or utensils with warm, soapy water.	Right now, it’s important to feed your baby peanut butter. Caregivers can try this when other children are at school or napping. If an allergic child touches peanut butter, they may have localized symptoms (hives, rash), but systemic allergic reactions and anaphylaxis occur due to *consumption*. If the caregiver needs additional support, consider offering two-bite feed introductions in the office for those with allergic family members. Caregivers must be educated to continue trying at home.
My baby is allergic to peanuts, and we have a family history of allergies to nuts. Can we test for nut allergies in addition to peanuts?	If testing before introduction makes the caregiver feel more comfortable, it can be an option. Additional testing may be a separate visit outside of the well child check, and take a bit longer to get results. We can schedule a different visit to discuss this.	Remember that false positives can lead to unnecessary avoidance, and we try not to medicalize the introduction of foods.
Isn’t peanut butter still a choking hazard for babies?	Yes! Mixing peanut butter with the suggested ingredients on the handout reduces the peanut butter’s thickness, making it a safe way to offer peanut butter without concerns for choking.	Whole nuts and nut fragments are choking hazards.
Does WIC (Women, Infants, and Children) cover peanut butter?	WIC only covers peanut butter before age 1 if the baby is breastfeeding.	-
What other peanut-containing foods are available besides peanut butter?	Some babies enjoy eating peanut ‘puffs.’ These are available online and in stores. Peanut powder is commercially available, and caregivers can mix 2 teaspoons like peanut butter.	Please note that babies must consume about 15-20 puffs, depending on the brand, to meet the target of 2 g of protein per serving.
What kind of peanut butter should we purchase?	We understand some peanut butter has sugar and other additives. It’s not necessary to buy a specific type of peanut butter or high-end organic peanut butter.	Try to buy peanut butter with as few ingredients as possible.
Is peanut allergy common or likely in my child’s race/ethnicity?	The rate of increase in self-reported food allergy is highest in Black children, followed by Hispanic children, and lowest in non-Hispanic white children.	The prevalence of food allergy in children over the last 20 years is about 8% (1 in 13 kids and 2 in every classroom are easily visualized statistics).
In our family/culture, we do not typically consume peanut butter. Does my baby still need to consume peanut butter?	Yes. Though your family may not offer peanut butter as a cultural norm, one of the safest ways for young children to eat peanut-containing foods is by thinning peanut butter (reference the handout with mixing directions).	Consistently offering your baby peanut butter during infancy and young childhood will allow them to consume other peanut products throughout their lives, as observed in various cultures.
Can we just go to an allergist first?	Our offices collaborate closely with our allergy colleagues. Referrals to allergists may delay the introduction of peanut-containing foods due to the wait time for new patient appointments.	Delaying the introduction of peanuts can actually increase the risk of developing a peanut allergy. We’re recommending an introduction to your baby's diet because delays in peanut introduction can increase the risk of developing peanut allergy.

EMR Changes

EMR note template changes included multi-select drop-down lists for introducing solid foods, eggs, and peanuts. The QI team provided a list of the smart data elements required to be included. Otherwise, providers’ individual note templates could retain a unique style and content. After feedback from provider champions during a PDSA cycle, the team modified the drop-down menu to include a Smart Phrase that accounted for children who were not yet developmentally ready for solid food introduction or who had other contraindications to solid food introduction. During a PDSA cycle, we highlighted the text of these important smart phrases in green to clarify for providers the elements of WCC encounters they should include in their notes, while still allowing them flexibility to organize the template as they wish. The team emphasized the importance of using these Smart Phrases verbatim, as their proper use directly feeds into the data reporting automation.

Data Reporting Automation

The pilot initiative relied exclusively on manual data review, which limits the feasibility of sustained efforts in future data analysis and PDSA cycle planning in response to performance decline with increasing sample sizes. Conversely, with the spread project, EMR analysts worked closely with the QI team's quality analyst. Together, they generated an analytics catalog report in the Epic dashboard, allowing participating clinics to access real-time data during implementation and sustainability, filtered by time, clinic location, and specific providers for outcome and process measures. This addition to the toolkit maintains the coding framework to ‘turn on’ other clinics within the healthcare system in the dashboard report if they want to adopt the initiative. The tool can also be shared across institutions with appropriate data-sharing permissions.

Planning for Sustainability

The project leadership and provider champions consolidated key information for new providers or clinics considering adopting the EPI toolkit. This compiled toolkit, or change package, includes written directions, screenshots, video examples of processes, and contact information. Additionally, provider champions presented the team’s interim QI data to stakeholders at physician-based clinics in other organizations, promoting the toolkit's dissemination to affiliated clinics. The capabilities of this analytics report enable clinics or individual providers to access and run reports or data analytics at any time for any provider(s) or clinic(s) that have adopted the smart data elements in their note templates.

Study of the Intervention

Data from baseline, the project design phase, implementation, and sustainability for four-, six-, nine-, and 12-month WCCs in the three outpatient clinics were compiled across seven PDSA cycles (Table 3).

**Table 3:** Plan-do-study-act (PDSA) cycles. Also known as rapid change cycles, PDSA cycles are a continual process of planning, testing, studying, and learning by comparing results with expected outcomes. Through repetitive iterations of the cycle and small real-time tests of change, quality improvement teams can quickly assess whether changes are having the desired effect by bringing the measured outcome closer to the intended goal [26,27]. AVS, after-visit summary; EMR, electronic medical record

Date	PDSA cycle	Changes to improve outcomes
1/8/2024	1	Distribution to Clinics 1 and 2 of a Frequently Asked Questions (FAQ) document to guide providers in addressing common family questions about early peanut introduction
1/24/2024	2	Implementation of vanishing and colored text on the template as a visual reminder to add the home peanut introduction handout to the AVS
1/29/2024	3	Feedback to clinic champions on provider adoption of new smart phrases
3/1/2024	4	Distribution to all clinics of free peanut jars to ensure equitable access to peanut butter for all patients
3/25/2024	5	Distribution of a video that demonstrates how providers can insert a home peanut introduction handout in AVS in response to feedback from providers that a video demonstration would supplement written instructions for Epic EMR
7/1/2024	6	Revise the wording in the smart data element to better accommodate varying infant readiness for solid food introduction, based on feedback from providers at the clinic champions meeting.
9/16/2024	7	CDS toolkit *change package* instructions made available on the intranet for new providers at existing participating practices or for new practices within the health system wishing to adopt the initiative after feedback from data presentation at the internal Children’s Health Committee

Measures and analysis

Table 4 illustrates the measures for this spread project. Our primary outcome measure focused on caregivers' reports of infant peanut consumption at the six-, nine-, and 12-month WCCs. Two process measures tracked provider documentation behaviors within the Epic EMR: Inserting the parent handout into the AVS and documenting EPI guidance at four-, six-, and nine-month WCCs. One balancing measure tracked patient in-room time, defined as the period from when patients were placed in a provider room until they checked out. The QI team used control charts for the measures analysis, observing the Shewhart Rules in determining clinical and statistical significance. The project leadership used QI Macros for Excel (KnowWare International Inc., Denver, CO; Version 2018) to generate statistical process control charts (SPCCs) and analyze data. We collected and reported data for infants seen in the three outpatient clinics for routine four-, six-, nine-, and 12-month WCCs over approximately 17 consecutive months. We excluded WCCs with twins.

**Table 4:** Project measures. SPCC, statistical process control chart; QI, quality improvement; WCC, well-child check; AVS, after-visit summary; EPI, early peanut introduction

Measure type	Description	Measure	Significance	Analysis
Outcome	Caregiver-reported peanut consumption by infants	Percent of six-, nine-, and 12-month WCCs with caregiver-reported consumption of peanuts by infants	Measurement of QI initiative on caregiver behavior in response to provider guidance	SPCC with statistical significance using Shewhart rule for significance
Process	EPI guidance from providers to patient caregivers	Percent of four-, six-, and nine-month WCCs with provider documentation of EPI guidance	Measurement of QI initiative on provider behavior	SPCC with statistical significance using Shewhart rule for significance
Process	Distribution of home peanut introduction handout	Percent of four-, six-, and nine-month WCCs with parent handout included in AVS	Measurement of QI initiative on provider behavior	SPCC with statistical significance using Shewhart rule for significance
Balancing	Patient time in-room (minutes)	Average visit length (minutes) for four-, six-, and nine-month WCCs (as measured by start time in patient room to time of checkout)	Measurement of QI impact on the length of visit, workflow, provider interaction	SPCC with statistical significance using Shewhart rule for significance

Ethical considerations

The Institutional Review Board (IRB) determined that this initiative does not constitute human subjects research as defined under federal regulations and does not require IRB approval (IRB# 23-2269).

## Results

**Figure 1:**
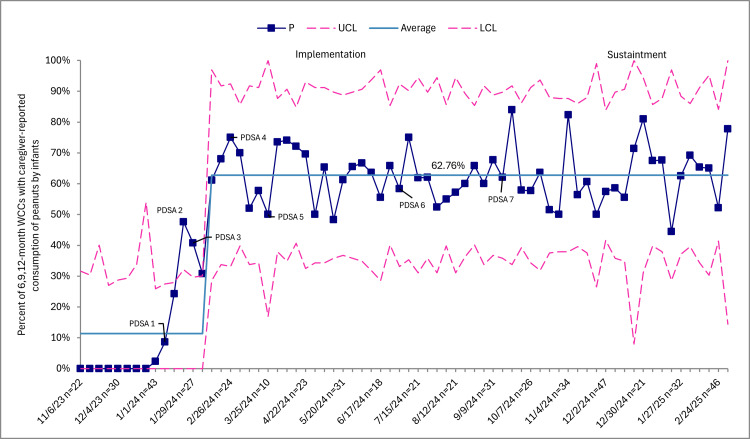
Caregiver-reported peanut consumption among infants, p-chart. Squares represent weekly data points in relation to the mean and upper control limits (UCLs) and lower control limits (LCLs). This control chart excludes individuals who are twins, diagnosed with a peanut allergy, have had an allergic reaction after trying peanuts, or are not ready for the introduction of solids. Baseline performance before toolkit implementation was 0%, reflecting the absence of standardized documentation tools to capture caregiver-reported peanut consumption. However, the initial centerline displayed in the chart is not zero because it includes early post-implementation data points. Consistent with statistical process control methodology, the centerline was recalculated only after sustained special cause variation indicated a new, stable level of performance. WCCs, well-child checks

**Figure 2:**
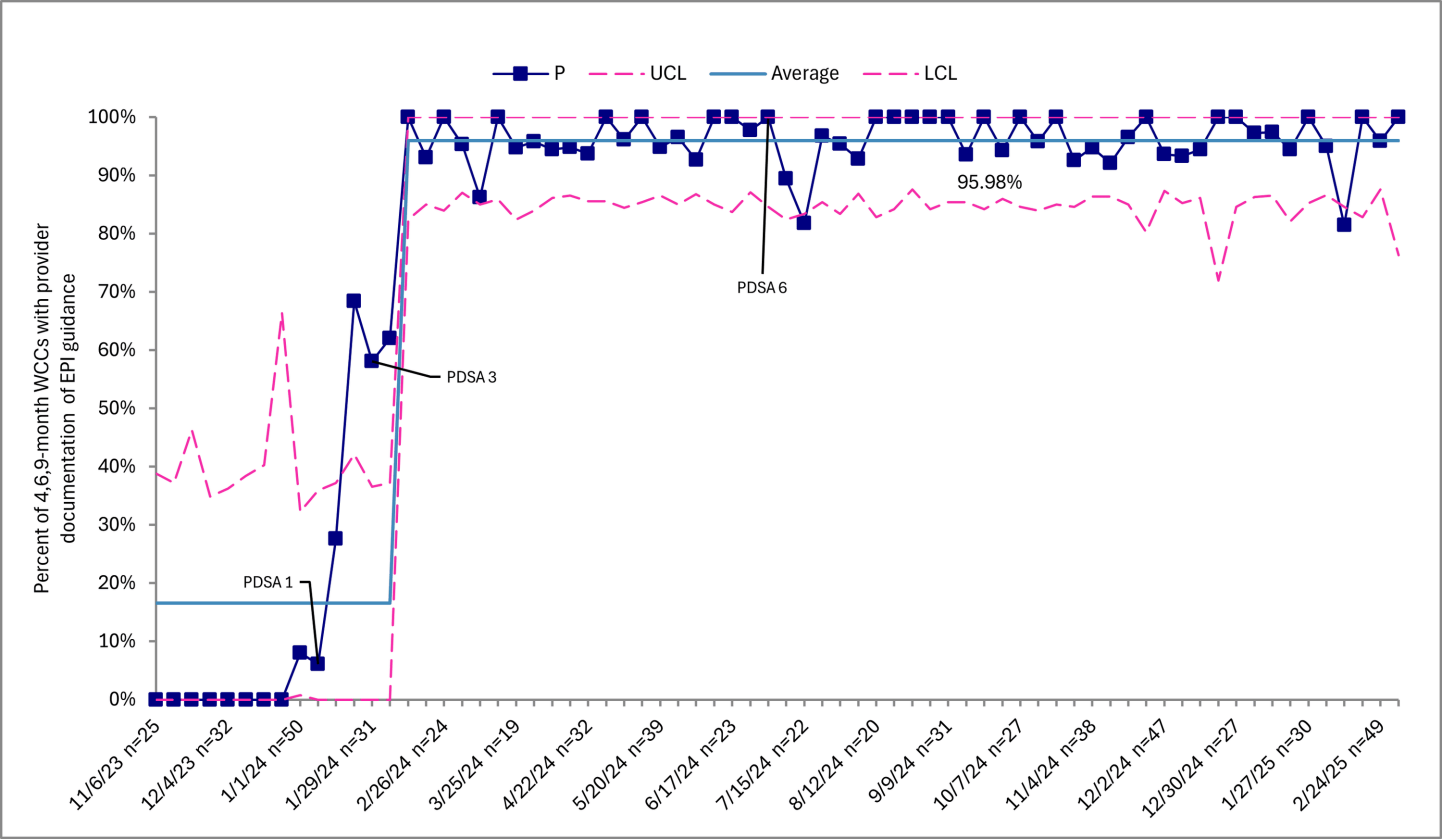
EPI guidance from providers to patient caregivers, p-chart. Squares represent weekly data points in relation to the mean and upper control limits (UCLs) and lower control limits (LCLs). This control chart excludes individuals who are twins, diagnosed with a peanut allergy, have had an allergic reaction after trying peanuts, or are not ready for the introduction of solids. Baseline performance before toolkit implementation was 0%, reflecting the absence of standardized documentation tools to capture caregiver-reported peanut consumption. However, the initial centerline displayed in the chart is not zero because it includes early post-implementation data points. Consistent with statistical process control methodology, the centerline was recalculated only after sustained special cause variation indicated a new, stable level of performance. WCCs, well-child checks; EPI, early peanut introduction

**Figure 3:**
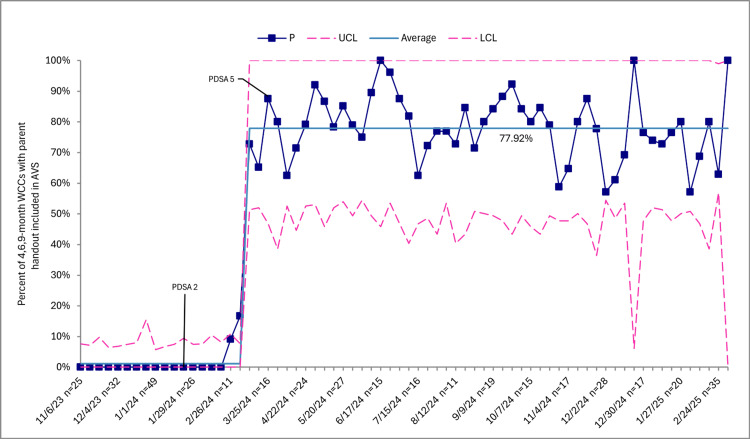
Distribution of home peanut introduction handout by clinic, p-chart. Squares represent weekly data points in relation to the mean and upper control limits (UCLs) and lower control limits (LCLs). This control chart excludes individuals who are twins, diagnosed with a peanut allergy, have had an allergic reaction after trying peanuts, are already eating peanuts, or are not ready for the introduction of solids. Baseline performance before toolkit implementation was 0%, reflecting the absence of standardized documentation tools to capture caregiver-reported peanut consumption. However, the initial centerline displayed in the chart is not zero because it includes early post-implementation data points. Consistent with statistical process control methodology, the centerline was recalculated only after sustained special cause variation indicated a new, stable level of performance. WCCs, well-child checks

Caregiver-reported peanut consumption during six-, nine-, and 12-month WCCs increased from 0 (0%) at baseline to 1,579 (62.8%) through sustainability (Figure 1). Documentation of EPI guidance in the Epic EMR note templates at four-, six-, and nine-month WCCs increased from 0 (0%) at baseline to 1,667 (96%) through sustainability (Figure 2). Distribution of the home peanut introduction handout in the patient’s AVS at four-, six-, and nine-month WCCs increased from 0 (0%) at baseline to 1,015 (77.9%) through sustainability (Figure 3). Lastly, the mean in-room time remained 48.8 minutes through sustainability.

SPCCs showed improvements in caregiver-reported peanut consumption, EPI guidance, and AVS handout distribution, reflecting special cause variation following implementation. Baseline performance before toolkit implementation was 0%, reflecting the absence of standardized documentation tools to capture caregiver-reported peanut consumption. The initial centerline included early post-implementation data points, which explains why the mean was not zero. However, consistent with SPCC rules, the centerline was not shifted immediately upon the first increase. Instead, it was recalculated only after evidence of sustained special cause variation and the establishment of a new, stable level of performance.

## Discussion

This QI project successfully increased EPI practices during WCCs at three hospital-affiliated clinics. Providers increased documentation of EPI counseling and the use of a home peanut introduction handout. Additionally, there was an increase in caregiver-reported peanut consumption rates at six, nine, and 12 months of age. Although many factors contribute to in-room time, our interventions did not appear to negatively affect this variable.

While outcome and process measure SPCCs have baseline values of 0%, it does not necessarily indicate that EPI conversations were not happening in practice before the launch of the intervention. Providers may have discussed EPI during WCCs, and families may have already been introducing peanuts at home. However, before the implementation of standardized documentation tools, there was no structured, reliable way to capture these conversations or caregiver-reported peanut consumption in the EMR. Outside of manual chart review, there was no consistent method to quantify or verify this activity. Therefore, the baseline reflects the absence of standardized documentation and measurement tools rather than the complete absence of EPI-related practice.

Documentation of EPI guidance SPCCs revealed two distinct out-of-control data points after the mean was shifted to 96% of providers' documented guidance. One of the pediatric clinics experienced a staffing change, with new faculty joining the practice and other faculty leaving around July 2024, which may explain the out-of-control data point and the decrease in documentation of EPI guidance by providers during this time. In discussions with the clinic champion, these providers, new to the practice, were acclimating to all aspects of relocation, including EMR documentation. We were encouraged to see a robust rebound in the data the following month, indicating that the system our clinic used to train new providers to use the CDS toolkit was effective. During the week of February 10, 2025, five visits were expected to include documentation of EPI guidance, but none did. Of the five visits, two infants had caregiver-reported peanut consumption documented by the provider. Therefore, it is possible that the providers perceived a redundancy in guiding these two infants, as they were already meeting the primary outcome. Reassuringly, we did not observe other unexplained out-of-control points in our processes.

Also notable was the gradual and sustained increase in caregiver-reported peanut consumption, our primary outcome measure. We interpret this pattern as expected early adoption following the toolkit rollout. Initially, only infants seen for targeted well-child visits after implementation would have received structured EPI guidance. By February 2024, approximately three months after our first kick-off meeting, more unique patients would have had at least one exposure to the EPI guidance and handout. As infants aged into subsequent WCCs, cumulative exposure to standardized counseling likely contributed to the continued upward trend. As our results relate to the timing of PDSA cycles, we saw data trending upward with cycles one, two, and three, but it was with PDSA cycles four through seven where the mean line shifted. These last PDSA cycles reflect a period of re-planning, rapid change, and re-testing of the CDS toolkit and EMR note templates based on provider feedback from in-clinic use of the tools. We believe the feedback from providers and the tailored response from the QI led to the desired outcome in these measures.

The increase in EPI documentation and caregiver-reported peanut consumption at home is meaningful because studies show that EPI in high-risk infants plays an important role in preventing the development of peanut allergies, and that protection against peanut allergy from EPI appears to last until at least adolescence [17,18]. Our project is situated in a clinical landscape where this benefit is known; however, many providers do not include this counseling in routine WCCs, and QI projects attempting to improve introduction have mixed results [3,7,9,12,16,30]. 

The initial pilot project, conducted in a pediatric residency continuity clinic setting, similarly showed improvement in EPI counseling and reported consumption by caregivers [7]. This spread QI project improved EPI counseling and caregiver reports of peanut consumption, even compared to the pilot project. This QI project not only expanded its reach both geographically and in the number of infants involved but also diversified the types of clinics hosting the intervention. While implementation challenges exist in a resident clinic, there is also generally a culture of QI that can readily adapt to change. The Epic EMR tools were successfully integrated across all clinics and adopted by providers.

In addition, the three clinics added diversity in settings, demonstrating that our tools effectively support clinicians across a range of settings and patient demographics. We suspect that the consistency of providers across clinics involved in the spread project enabled better education about the CDS toolkit and easier integration into existing WCC frameworks. Additionally, the new dashboard report allowed more immediate feedback from the clinic QI project champions to individual providers.

Selecting three different clinics ensured that the CDS toolkit continued to perform well for users across three distinct primary care settings. Having an automated analytics report tool was an innovative addition, which could allow other clinics within the current healthcare system to adopt the dashboard reporting feature by following stepwise instructions left in the QI team’s *change package* and communicating with the healthcare entity’s Epic team. The automated reporting dashboard was designed with the sustainability vision of broad expansion within and to other Epic EMR-capable health systems. Data collection in the pilot project was manual, which is not a sustainable method for expanded projects such as this and other spread QI initiatives. Data-sharing agreements between our institution and other Epic users could be strengthened by sharing the code for this automated reporting tool. We focused on a single allergen (peanut) and measures associated with this project. Still, the proof of concept has significant implications for developing robust tools to address the unmet need to introduce other allergenic foods to young infants, thereby continuing allergy prevention efforts.

Our balancing measure, time-in-room, showed no special cause variation across the individual clinics. One of the barriers to adding counseling about EPI at WCCs is the already plentiful counseling recommended by professional societies [20,21]. While our project found that patients' time in the room did not increase, further investigation is needed to understand how discussions of other aspects of preventive counseling changed during these visits, which represents a limitation.

An additional limitation is that, while integrating tools into the EMR was a strength in our clinical settings, it would pose a barrier for clinics that rely on paper records or an EMR with limited customization. There is a need for informatics scientists to create a similar EMR architecture for other user systems to generate data reporting tools to further disseminate this type of initiative. Another limitation is that although our project captured caregiver-reported peanut consumption, we were unable to track actual rates of peanut allergy development due to the relatively low prevalence of peanut allergy. A retrospective chart review of infants included in the pilot and spread project in the coming years may further justify past and ongoing QI efforts if we find that peanut allergy rates in our population are lower than the national rate of childhood peanut allergy. Notably, the primary outcome measure was based on self-reports from caregivers, and this can be considered a limitation compared to peanut consumption that could be documented or observed by members of the QI team or other clinicians.

Lastly, future research on EPI and early allergen introduction should identify gaps in health equity by examining how perceptions of EPI influence providers and patient caregivers from diverse demographic backgrounds. Improving guideline adherence through QI efforts could reduce the need for allergy referrals and lower peanut and other food allergy rates across a more representative sample of our community and population.

## Conclusions

EPI remains necessary to reduce peanut allergy rates among young children nationwide. EPI can be effectively integrated into WCCs across various primary care settings through EMR documentation and standardized tools. Educational guidance provided to families can improve reported peanut consumption rates without increasing in-room visit time. Broad adoption of standardized CDS toolkits across primary care settings will improve evidence-based education on peanut introduction and provide a template for discussing the introduction of all allergenic foods in infancy.

Reporting dashboards and provider education should be used alongside a well-established foundation of methodically tested and proven CDS tools. As clinicians also aim to increase rates of consumption of other allergenic foods in infancy, QI projects may develop comprehensive CDS toolkits, coupled with a tracking and reporting tool, as we demonstrated in this project.

## References

[1] Togias A, Cooper SF, Acebal ML (2017). Addendum guidelines for the prevention of peanut allergy in the United States: Report of the National Institute of Allergy and Infectious Diseases-sponsored expert panel. J Allergy Clin Immunol.

[2] Giovannini M, Bolis M, Barni S (2023). Pearls and pitfalls of weaning an infant with severe atopic dermatitis and sensitization/allergy to food. J Clin Med.

[3] Bilaver LA, Martusiewicz MN, Jiang J, Gupta RS (2019). Effectiveness of clinical decision support tools on pediatrician adherence to peanut allergy prevention guidelines. JAMA Pediatr.

[4] Greenhawt M, Chan ES, Fleischer DM (2018). Caregiver and expecting caregiver support for early peanut introduction guidelines. Ann Allergy Asthma Immunol.

[5] Gupta RS, Bilaver LA, Johnson JL (2020). Assessment of pediatrician awareness and implementation of the addendum guidelines for the prevention of peanut allergy in the United States. JAMA Netw Open.

[6] Lieberman JA, Gupta RS, Knibb RC, Haselkorn T, Tilles S, Mack DP, Pouessel G (2021). The global burden of illness of peanut allergy: a comprehensive literature review. Allergy.

[7] Herlihy LE, Walters EM, D'Auria JP, Orgel K, Jordan KA (2023). Early peanut introduction in infants: Improving guideline adherence with EMR Standardization. Pediatrics.

[8] Fleischer DM, Chan ES, Venter C (2021). A consensus approach to the primary prevention of food allergy through nutrition: Guidance from the American Academy of Allergy, Asthma, and Immunology; American College of Allergy, Asthma, and Immunology; and the Canadian Society for Allergy and Clinical Immunology. J Allergy Clin Immunol Pract.

[9] Anagnostou A, Lieberman J, Greenhawt M (2023). The future of food allergy: challenging existing paradigms of clinical practice. Allergy.

[10] Greer FR, Sicherer SH, Wesley Burks A (2019). The effects of early nutritional interventions on the development of atopic disease in infants and children: the role of maternal dietary restriction, breastfeeding, hydrolyzed formulas, and timing of introduction of allergenic complementary foods. Pediatrics.

[11] Obbagy JE, English LK, Wong YP (2019). Complementary feeding and food allergy, atopic dermatitis/eczema, asthma, and allergic rhinitis: a systematic review. Am J Clin Nutr.

[12] Abrams EM, Shaker M, Greenhawt M, Mack DP (2022). International peanut allergy prevention, 6 years after the learning early about peanut study. J Allergy Clin Immunol Pract.

[13] Tapke D, Prince B, Scherzer R, Stukus D, Mikhail I (2021). A retrospective cohort study of pediatrician implementation of the 2017 United States early peanut introduction guidelines. Ann Allergy Asthma Immunol.

[14] Abrams EM, Brough HA, Keet C (2020). Pros and cons of pre-emptive screening programmes before peanut introduction in infancy. Lancet Child Adolesc Health.

[15] Johnson JL, Gupta RS, Bilaver LA (2020). Implementation of the addendum guidelines for peanut allergy prevention by US allergists, a survey conducted by the NIAID, in collaboration with the AAAAI. J Allergy Clin Immunol.

[16] Pitts MA, Sashidhar S, Hudak P, Blood-Siegfried J (2020). Early peanut protein introduction in clinical practice. J Pediatr Nurs.

[17] Du Toit G, Roberts G, Sayre PH (2015). Randomized trial of peanut consumption in infants at risk for peanut allergy. N Engl J Med.

[18] Du Toit G, Huffaker MF, Radulovic S (2024). Follow-up to adolescence after early peanut introduction for allergy prevention. NEJM Evid.

[19] Lo RM, Purington N, McGhee SA, Mathur MB, Shaw GM, Schroeder AR (2021). Infant allergy testing and food allergy diagnoses before and after guidelines for early peanut introduction. J Allergy Clin Immunol Pract.

[20] (2024). 2024 Recommendations for preventive pediatric health care: policy statement. Pediatrics.

[21] Turner K (2018). Well-child visits for infants and young children. Am Fam Physician.

[22] Lander J, Bitzer EM, von Sommoggy J (2023). How do parents access, appraise, and apply health information on early childhood allergy prevention? A focus group and interview study. Front Public Health.

[23] Lai M, Sicherer SH (2019). Pediatricians underestimate parent receptiveness to early peanut introduction. Ann Allergy Asthma Immunol.

[24] Rowland AF, Nguyen TH, Cunha PP, Ezhuthachan I, Orenstein E, Kandaswamy S, Lee T (2024). Implementing a clinical decision support tool to increase early peanut introduction guidance. J Allergy Clin Immunol.

[25] Nguyen TH, Cunha PP, Rowland AF, Orenstein E, Lee T, Kandaswamy S (2023). User-centered design and evaluation of clinical decision support to improve early peanut introduction: formative study. JMIR Form Res.

[26] Russ SJ, Green J, de Winter L (2023). An introduction to quality improvement. J Clin Urol.

[27] McQuillan RF, Silver SA, Harel Z (2016). How to measure and interpret quality improvement data. Clin J Am Soc Nephrol.

[28] Abrams EM, Ben-Shoshan M, Protudjer JL, Lavine E, Chan ES (2023). Early introduction is not enough: CSACI statement on the importance of ongoing regular ingestion as a means of food allergy prevention. Allergy Asthma Clin Immunol.

[29] Roberts G, Bahnson HT, Du Toit G (2023). Defining the window of opportunity and target populations to prevent peanut allergy. J Allergy Clin Immunol.

[30] Hauk L, Volerman A, Cifu AS (2019). Increasing discussions on early peanut introduction- a quality improvement project. Eur J Allergy Clin Immunol.

